# Identifying factors and predicting mental health issues in polypharmacy elderly using machine learning: a study based on the English longitudinal study of aging

**DOI:** 10.3389/fpsyg.2026.1773958

**Published:** 2026-05-15

**Authors:** Hongju Wang, Fangqing Xie, Yu Wu, Yan Zhou, Xirui Guo, Shihao Yan, Hua Wei, Shibo Lin, Fang Yang, Chun Liu

**Affiliations:** 1 Department of Pharmacy, West China School of Medicine, Sichuan University, Sichuan University Affiliated Chengdu Second People's Hospital, Chengdu Second People's Hospital, Chengdu, Sichuan, China; 2 College of Traditional Chinese Medicine, Jilin Agricultural University, Changchun, Jilin, China

**Keywords:** elderly, KNN model, machine learning, mental health, polypharmacy, SHAP

## Abstract

**Objectives:**

Polypharmacy is common among the elderly and is associated with various health issues. Identifying risk factors and developing accurate predictive models for mental health issues in polypharmacy elderly individuals is crucial for early intervention.

**Methods:**

Data from the English Longitudinal Study of Aging (ELSA) were used, covering waves 1–9. The study identified factors associated with mental health issues through clinical baseline analysis and univariate logistic regression. Twelve machine learning models were constructed and validated using the mlr3 framework, with KNN emerging as the optimal model based on AUC, DCA, and calibration curves. SHapley Additive exPlanations (SHAP) analysis was used to interpret the KNN model.

**Results:**

Baseline analysis and logistic regression identified antidepressant use, age, pain frequency, smoking history, and respiratory improvement medication use as significant factors. The KNN model demonstrated superior performance with an AUC of 0.901 in the training set and 0.827 in the validation set, showing high discrimination ability and robustness.

**Conclusion:**

This study identified key factors associated with mental health issues in polypharmacy elderly and validated the KNN model as an effective predictive tool. These findings can inform targeted interventions and improve mental health outcomes in this vulnerable population.

## Introduction

Mental health issues among the elderly are becoming increasingly severe. Research indicates that approximately 20% of people over the age of 55 are affected by various mental disorders, with anxiety, cognitive impairment, and mood disorders being the most common (Petrova and Khvostikova, 2021). However, due to factors such as stigma, misunderstanding of the disease (such as mistaking it for “normal aging”), and insufficient medical resources, there is a significant treatment gap. Data shows that only about half of the patients report having psychological problems, and the proportion of those who eventually receive professional mental health services is extremely low (Caspi et al., 2024). The diagnosis of mental disorders in the elderly presents unique challenges. Elderly people often have multiple chronic physical diseases, and their symptoms (such as fatigue, insomnia, and cognitive decline) overlap highly with those of mental disorders like depression and anxiety, increasing the difficulty of differential diagnosis, additionally, cognitive impairment or cultural factors make it difficult for some patients to accurately express their emotional distress (Devita et al., 2022).

With the intensification of global population aging, polypharmacy has become an important issue in geriatric medicine and geriatric psychiatry. Polypharmacy typically refers to the concurrent use of five or more medications, including prescription drugs, over-the-counter drugs, and dietary supplements (Masnoon et al., 2017). Among the elderly, the phenomenon of polypharmacy is particularly common due to the frequent coexistence of multiple chronic diseases. There exists a complex and bidirectional relationship between polypharmacy and geriatric mental health. On the one hand, polypharmacy is an important risk factor for geriatric mental problems. Studies have shown that the risk of mild cognitive impairment in the elderly who use five or more drugs increases by 32%−37% (Molino et al., 2025). Certain drugs (such as those with anticholinergic properties) or drug interactions may directly lead to cognitive decline, mood disorders (such as increased depression or anxiety), and even induce delirium, hallucinations, and other psychotic symptoms. For instance, some cardiovascular drugs and sedative-hypnotics can interfere with emotional stability by affecting the neurotransmitter system (López-Álvarez et al., 2019). On the other hand, geriatric mental problems can also further promote the occurrence of polypharmacy. Clinically, to control complex or intractable mental and behavioral symptoms (such as insomnia, anxiety, and agitation), doctors may combine the use of multiple psychiatric drugs. Additionally, elderly patients with mental disorders often coexist with multiple physical diseases (such as hypertension, diabetes, and metabolic syndrome), requiring concurrent treatment with multiple medications, further exacerbating the burden of medication use (de Lima et al., 2020; Stuhec, 2022). A population-based study in Canada pointed out that the proportion of polypharmacy (especially the combined use of multiple psychiatric drugs) among elderly patients with schizophrenia has been continuously increasing, often accompanied by an increase in the use of medications for metabolic diseases (Lunghi et al., 2023). However, existing research mostly focuses on the analysis of medication use for specific diseases (such as dementia), lacking systematic exploration of the macroscopic association and long-term impact between polypharmacy and mental health outcomes.

This study utilized the data from the UK Longitudinal Study on Aging (ELSA) to link the data on polypharmacy and mental disorders. This research identified the key factors related to the mental health problems of elderly patients treated with multiple medications, and through the construction of 12 machine learning algorithms, starting from different paradigms and underlying principles such as linear, non-linear, ensemble, and deep learning, it conducted multi-angle diagnostic model construction and verified that the KNN model served as an effective predictive tool. These findings can provide information for targeted intervention measures and improve the mental health outcomes of this vulnerable population.

## Materials and methods

### Data source

The data for this study were obtained from the English Longitudinal Study of Aging (ELSA) database, covering relevant data from wave 1 to wave 9. We adopted a cross-sectional analytical approach by pooling individual records from multiple waves. The dependent variable in this study was the physician diagnosis information regarding geriatric emotional, neurological, or psychiatric problems (psyche). The study collected information on patients' use of medications for hypertension (rxhibp), diabetes (rxdiabo, rxdiab), chronic pulmonary disease (rxlung), asthma (rxasthma), blood thinning (rxbldthn), osteoporosis (rxosteo), high cholesterol (rxhchol), and depression (rxdepres). Additionally, respiratory improvement medication use was identified using the fields (puffinhl, puffinhl_e). Based on the aforementioned medication information, polypharmacy was defined as the concurrent use of five or more medication categories. Given the constraints of a large-scale population survey, medications were aggregated by therapeutic class rather than individual pills, which serves as a validated proxy for total medication burden in epidemiological studies (Masnoon et al., 2017; Viktil et al., 2007; Prithviraj et al., 2012; Rasu et al., 2014). This study employed a cross-sectional study design. Polypharmacy and physician-diagnosed mental health issues were assessed based on self-reported and clinically verified records collected by trained nurses during standardized interviews, which were further coded using the British National Formulary (BNF) system to ensure consistency. This design allows us to identify the snapshot association between medication burden and mental health status in the elderly population. The initial population included individuals aged 50 years and above. To ensure data integrity regarding polypharmacy status and mental health outcomes, participants were excluded if they had missing data on medication use (preventing classification of polypharmacy) or missing outcome variable information. Furthermore, to effectively control for confounding factors and enhance the accuracy, interpretability, and generalizability of the clinical model, thereby reducing bias, this study included several covariates, such as age, gender, educational level, frequency and severity of pain, post-tax personal income, activity frequency, history of falls, smoking history, and drinking history. After applying the inclusion and exclusion criteria, the final analytical sample consisted of 355 elderly individuals with polypharmacy problems. The age range of the participants was 50–90 years, with a mean age of 70.4 years (median: 70 years). The sex distribution was balanced, comprising 170 males (47.9%) and 185 females (52.1%).

### Data preprocessing

To ensure the scientific rigor and reliability of the study conclusions, we first conducted a strict screening of the data, excluding all samples with missing dependent variable information. On this basis, we used the random forest algorithm to impute missing data. Using complete data as the target variable, we trained the random forest model and optimized the model parameters through multiple iterations to improve the accuracy of imputation. This method fully utilized the complex relationships between variables to predict missing values, thereby minimizing the bias introduced by incorrect estimation of missing values. After data standardization, we included the clinical information of 355 elderly individuals with polypharmacy problems in the study, randomly dividing them into a training set and a validation set in a 7:3 ratio. The training set was used for the formal analysis process and the construction of 12 machine learning models, while the validation set was used for the validation of all models and the identification of the optimal model.

### Clinical baseline analysis

We first assessed the distribution characteristics of all independent variables between individuals with and without geriatric emotional, neurological, or psychiatric problems, analyzing their basic data characteristics and statistical differences. For continuous variables, we used mean ± standard deviation for description and performed intergroup comparisons using the independent samples *t*-test; for categorical variables, we calculated the frequency and percentage of each category and performed intergroup comparisons using the χ^2^ test. Finally, we drew a table of baseline characteristics based on the above statistical analysis results. In all comparisons, a *P*-value of less than 0.05 was used as the criterion to determine that the variable had a significant intergroup difference.

### Univariate logistic regression analysis

To further screen variables with a relatively clear correlation with the dependent variable, we used univariate logistic regression analysis. We calculated the odds ratio (OR) for each variable to assess whether its impact on the dependent variable was statistically significant and to determine whether it was a risk factor or a protective factor for the dependent variable. Specifically, variables with *P* < 0.05 and *OR* ≠ 1 were considered to have a significant effect. Finally, all variables with significant effects in both the clinical baseline analysis and the univariate logistic regression analysis were included in the subsequent analysis for further machine learning model construction. Through this dual screening mechanism, we more comprehensively evaluated the quality and correlation of the variables, effectively reducing the number of variables entering the subsequent multivariate models, thereby improving the accuracy and stability of the models.

### Construction of the optimal machine learning algorithm prediction model

To address the complex, non-linear interactions often present in geriatric pharmacotherapy and mental health data, we employed a comparative modeling strategy. Rather than relying on a single algorithm, we constructed 12 models spanning linear, non-linear, ensemble, and deep learning paradigms. Includes multivariable logistic regression, ridge regression, LASSO regression, elastic net, support vector machine (SVM) with radial basis function (RBF) kernel, SVM with polynomial kernel, XGBoost, feedforward neural network (nnet), LightGBM, K-nearest neighbors (KNN), and recursive partitioning and regression tree (rpart) algorithms. These algorithms modeled the data from different perspectives to achieve the best prediction performance.

To ensure that each algorithm constructs the optimal model and forms a unified and comparable technical implementation framework, this study used the mlr3 machine learning model construction system, including the “mlr3” package (version 1.0.0), “mlr3tuning” package (version 0.6.0), “mlr3learners” package (version 0.12.0), and “mlr3extralearners” package (version 1.1.0.9000) (Qi et al., 2025; Wang et al., 2024). A grid search strategy was used for hyperparameter tuning analysis for each algorithm to determine the optimal hyperparameters. Under the optimal hyperparameters, a 3-fold cross-validation internal resampling strategy was used to complete model construction, thereby maximizing model performance and controllable generalization error.

Subsequently, based on the training set and validation set, ROC curves were drawn and the area under the curve (AUC) was calculated using the “pROC” package (version 1.18.5), decision curve analysis (DCA) curves were drawn using the “rmda” package (version 1.6), calibration curves were drawn using the “CalibrationCurves” package (version 2.0.7), and the net reclassification improvement (NRI) and integrated discrimination improvement (IDI) between models were calculated using the “PredictABEL” package (version 1.2-4) to comprehensively evaluate the objective performance, versatility, and robustness of all models in a unified manner, thereby identifying the optimal model (Robin et al., 2011).

### Interpretation of the optimal machine learning prediction model

To thoroughly analyze the identified optimal machine learning prediction model, this study conducted permutation feature importance (PFI) analysis using the “iml” package (version 0.11.4) and drew partial dependence-individual conditional expectation (PD-ICE) curves. Permutation feature importance analysis can objectively quantify the importance weights of each feature in the model and reveal its contribution to model prediction performance. The PD-ICE curves can intuitively display the non-linear relationship between features and model prediction results, as well as the trends in model predictions at different feature levels. In addition, SHapley Additive exPlanations (SHAP) analysis was further used to accurately quantify the marginal contribution of each feature to the model's single prediction or overall prediction results, thereby providing a more detailed and nuanced perspective for model interpretation (Cao and Hu, 2024).

## Results

### Identification of factors associated with mental health issues in polypharmacy elderly

**Figure 1:**
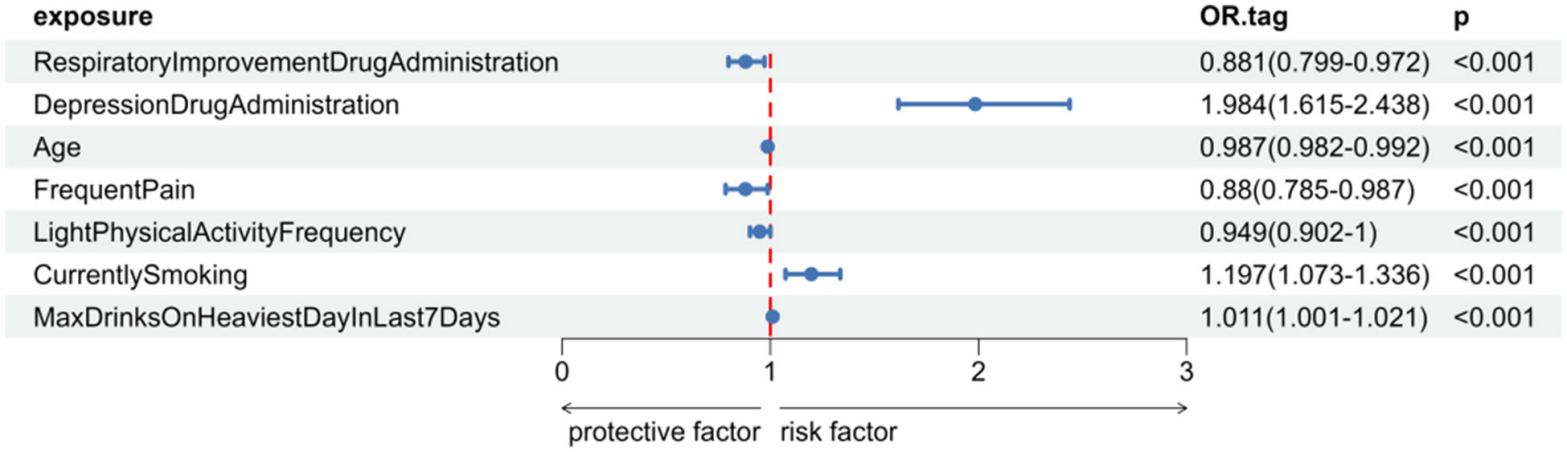
Univariate logistic regression of candidate predictors for mental-health issues in polypharmacy elderly.

The baseline analysis results showed that there were significant statistical differences in the use of respiratory improvement medications, antidepressant use, age, pain frequency, and smoking history between polypharmacy elderly individuals with and without mental health issues (Table 1). Among these factors, antidepressant use and age had the most significant differences between groups (*p* < 0.001). Univariate logistic regression analysis further revealed that antidepressant use, smoking frequency, and maximum recent alcohol consumption were risk factors for mental health issues in polypharmacy elderly individuals, while the use of respiratory improvement medications, age, pain frequency, and frequency of light physical exercise were protective factors (Figure 1). Based on the results of the baseline analysis and univariate logistic regression analysis, this study ultimately identified the use of respiratory improvement medications, antidepressant use, age, pain frequency, and smoking history as factors associated with mental health issues in polypharmacy elderly individuals (Figure S1).

**Table 1:** The baseline analysis results.

Name	Desc	0 (*N* = 200)	1 (*N* = 49)	Id	*P*
Respiratory improvement drug administration	0	99 (49.5%)	34 (69.4%)	Respiratory improvement drug administration 0	0.019
	1	101 (50.5%)	15 (30.6%)	Respiratory improvement drug administration 1	
Depression drug administration	0	198 (99%)	38 (77.6%)	Depression drug administration 0	< 0.001
	1	2 (1%)	11 (22.4%)	Depression drug administration 1	
Age	Mean ±*SD*	71.5 ± 8.5	65.1 ± 8.2	Age	< 0.001
Frequent pain	1	146 (73%)	43 (87.8%)	Frequent pain 1	0.048
	0	54 (27%)	6 (12.2%)	Frequent pain 0	
Currently smoking	0	155 (77.5%)	27 (55.1%)	Currently smoking 0	0.003
	1	45 (22.5%)	22 (44.9%)	Currently smoking 1	

### Determination of the optimal machine learning prediction model

**Figure 2:**
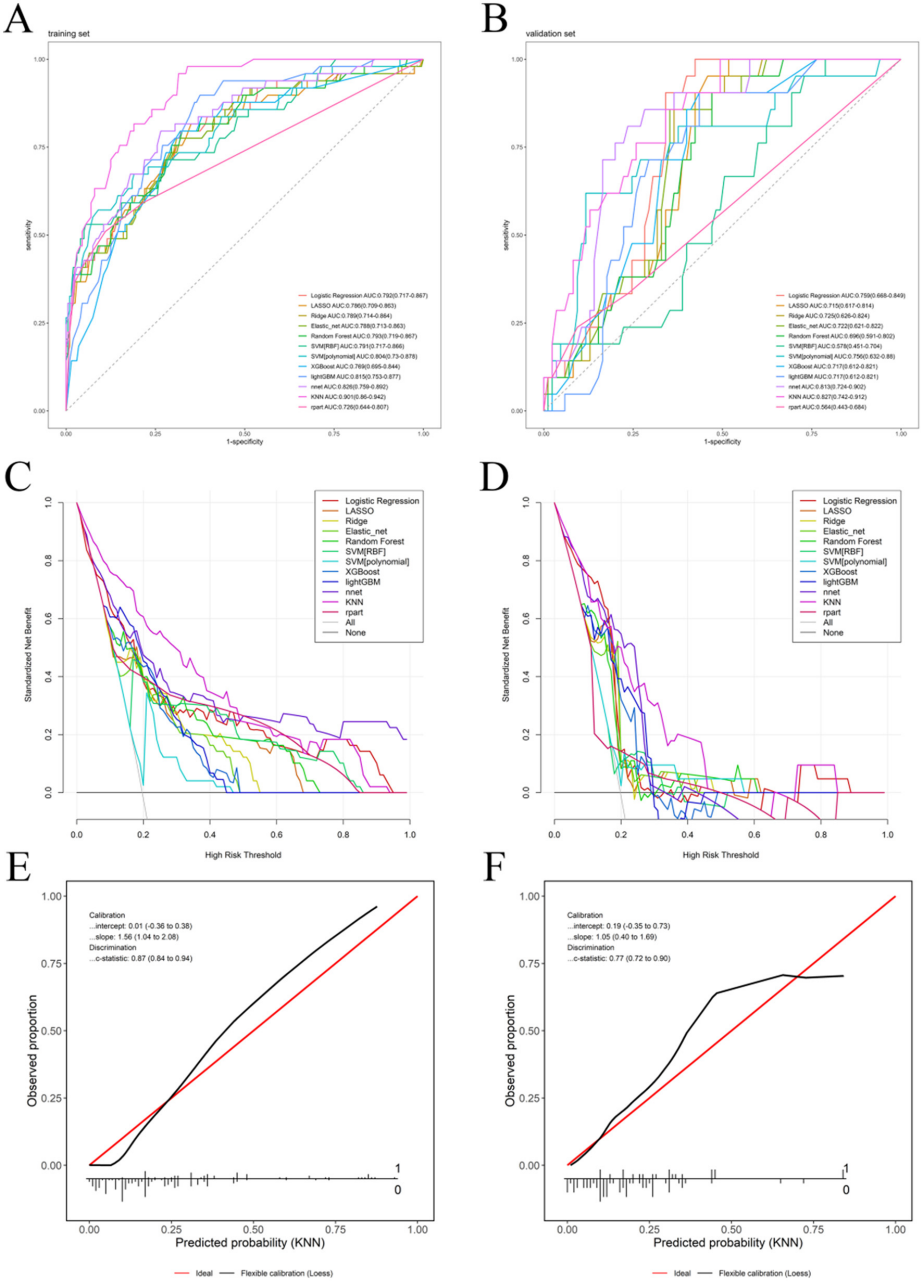
Performance comparison of 12 machine-learning models trained on five selected features. **(A)** ROC–training set (KNN AUC = 0.901), **(B)** ROC–validation set (KNN AUC = 0.827), **(C)** decision curve analysis (DCA)–training set, **(D)** DCA–validation set, **(E)** calibration–training set (*C*-statistic = 0.87), **(F)** calibration–validation set (*C*-statistic = 0.77).

Using the five identified factors associated with mental health issues in polypharmacy elderly as features, we constructed 12 machine learning prediction models under optimal hyperparameter conditions based on the mlr3 framework and performed unified validation and selection from multiple levels. The ROC validation curve of the training set showed that the AUC values of all models were above 0.7, with the KNN model having the highest AUC value of 0.901 (Figure 2A). The ROC curve of the validation set showed a similar trend, with all models except the random forest, SVM (RBF), and rpart models having AUC values above 0.7, and the KNN model still having the highest AUC value of 0.827 (Figure 2B). The DCA curves indicated that the KNN model performed well in both the training and validation sets, achieving the highest net benefit across most risk thresholds (Figures 2C, D). The calibration curves showed that the KNN model had small Intercept and Slope values, indicating low systematic bias; moreover, the KNN model had a *C*-statistic of 0.87 in the training set and 0.77 in the validation set, suggesting good discrimination ability, versatility, and robustness (Figures 2E, F). Additionally, we calculated the NRI and IDI between all models and drew a directed weighted network diagram, using the logrank algorithm to statistically determine the optimal model under the two evaluation rules. The results showed that, in both the training and validation sets, the KNN model had the highest cumulative NRI and IDI, further indicating that the KNN model was the best model (Figure S2). Based on all the validation results mentioned above, this study ultimately determined that, among the 12 machine learning prediction models, the KNN model was the optimal model.

### Interpretation of the KNN model

**Figure 3:**
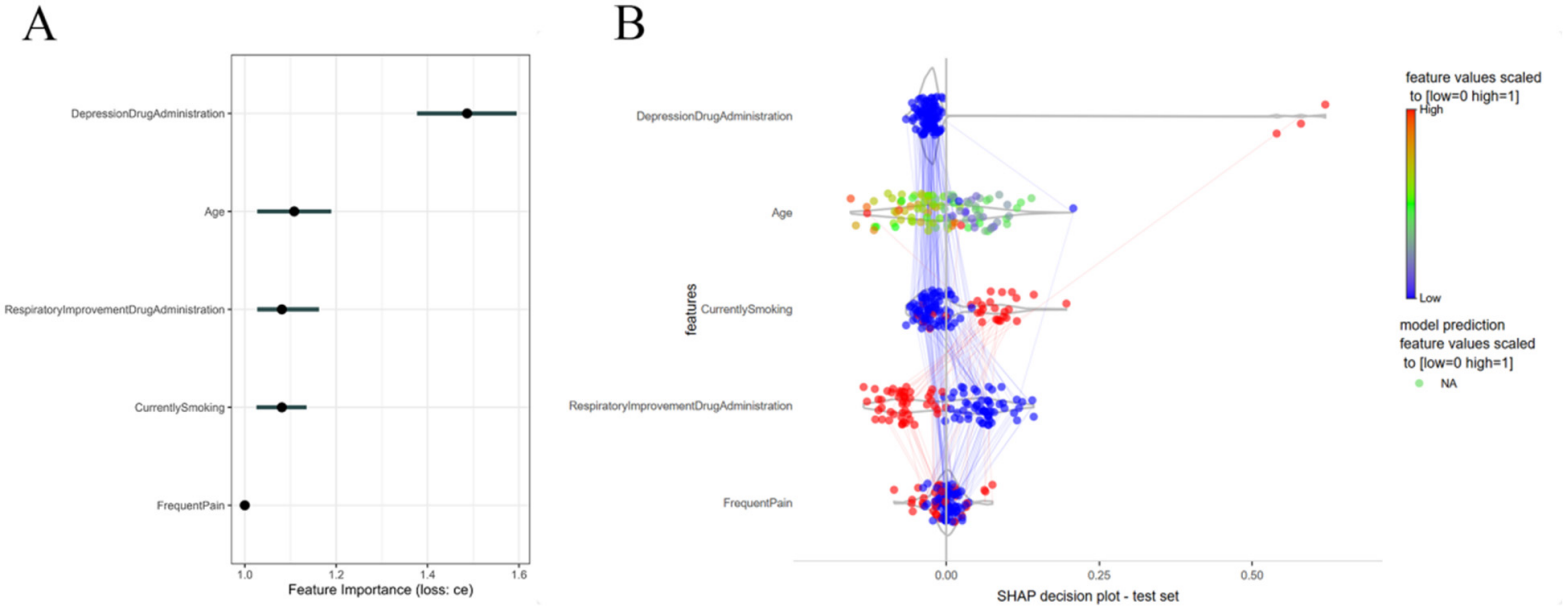
KNN model interpretation. **(A)** Permutation feature importance (PFI) ranking; **(B)** SHAP summary plot.

PFI analysis was employed in this study to interpret the KNN model. The results indicated that, within the KNN model, the feature importance of antidepressant use was the highest, followed by age, the use of respiratory improvement medications, smoking history, and pain frequency (Figure 3A). The slopes of the Partial Dependence-Individual Conditional Expectation (PD-ICE) curves were consistent with the PFI estimation trends, further elucidating the correlations between each feature and the dependent variable, which were largely in agreement with the conclusions drawn from the univariate logistic regression analysis (Figure S3). Moreover, SHAP analysis provided a more nuanced interpretation of the contribution of each feature to the final estimation results at the sample level, with overall trends consistent with the PFI analysis (Figure 3B).

## Discussion

This study identified that the use of antidepressants, age, pain frequency, smoking history, and the use of respiratory improvement drugs were significantly associated with mental illness. The key factors related to the mental health problems of elderly people on polypharmacy were determined, and the KNN model was verified as an effective predictive tool. These findings can inform targeted intervention measures and improve the mental health outcomes of this vulnerable population.

Building on the identification of antidepressant use as a key predictor, we first discuss its dual role in mental vulnerability. DepressionDrugAdministration usually implies that the patient has been clinically diagnosed with depression, and depressive disorder itself is one of the most common mental problems in old age. More concerning is that antidepressants themselves may directly cause or exacerbate certain mental symptoms. Multiple studies have found that the use of SSRI antidepressants is associated with cognitive decline and an increased risk of dementia in the elderly, with the risk being particularly pronounced for drugs with strong anticholinergic side effects (such as paroxetine) (Coupland et al., 2011). The mental side effects of antidepressants are further amplified in the context of polypharmacy, especially when used in combination with benzodiazepines, anticholinergic drugs, or antipsychotic drugs, significantly increasing the risk of central nervous system side effects of antidepressants (Gribbin et al., 2011). Antidepressants are not only the primary treatment for depression in the elderly population, but they may also act as an amplifier or trigger for mental problems. The results of this study suggest that “DepressionDrugAdministration” is not merely a drug variable, but rather a comprehensive indicator of “mental vulnerability”.

Beyond medication-related risk factors, lifestyle behaviors such as smoking also contribute substantially to mental health disorders in later life. This study confirmed that CurrentlySmoking is an independent risk factor for mental problems in the elderly. From the perspective of neurobiological mechanisms, smoking affects brain function through multiple pathways: Acute nicotine stimulation increases dopamine release in the mesolimbic system, and long-term use leads to down-regulation of dopamine receptors, increasing the risk of depression, anxiety and cognitive fluctuations; Chronic smoking reduces the level of brain-derived neurotrophic factor (BDNF), and BDNF deficiency is closely related to depression and cognitive decline in the elderly; Heavy metals and free radicals in tobacco induce chronic neuroinflammation, promote amyloid protein deposition, and accelerate the progression of cognitive impairment to dementia (Asharani et al., 2020; Wang et al., 2016). A study in Qatar found that the prevalence of psychotic disorders was significantly higher among current smokers (Zolezzi et al., 2022). Numerous drug interactions exist with smoking: accelerates the metabolism of antidepressants and antipsychotics, leading to dose escalation and accumulation of central nervous system toxicity (Carrillo et al., 2003; Li and Shi, 2015).

In addition to behavioral and pharmacological factors, physical comorbidities and corresponding medications also shape mental health in complex ways. Among the elderly population, mental problems often coexist with chronic physical diseases to a high degree. In particular, respiratory system diseases such as chronic obstructive pulmonary disease (COPD) and asthma have a significant bidirectional association with mental disorders such as depression and anxiety. This study found that RespiratoryImproveDrugAdministration has a significant protective effect on mental problems in the elderly. This finding is highly consistent with the research evidence in the field of respiratory-spiritual comorbidity (Global Initiative for Chronic Obstructive Lung Disease, 2023). Studies have shown that the prevalence of depressive symptoms in COPD patients is significantly higher than that in healthy individuals, and the anxiety prevalence in COPD patients is as high as 40%, with the panic disorder risk being 10 times that of the general population (Panagioti et al., 2014). Dyspnea is not only the core symptom of COPD but also an independent predictor of depressive symptoms. Its severity is positively correlated with the Hamilton Depression Rating Scale (HAMD-17) score (Vozoris et al., 2018). Respiratory function decline triggers or aggravates mental symptoms through various mechanisms such as hypoxemia, hypercapnia, systemic inflammation, and sleep disorders (Rahi et al., 2023; Yu et al., 2025; Siraj, 2025). RespiratoryImproveDrugAdministration exerts mental protection effects by improving cerebral tissue oxygenation, reducing systemic inflammation, improving sleep quality, enhancing activity ability, and self-efficacy (Stoller et al., 2010; Pumar et al., 2014; Simon et al., 2023; Koulouris et al., 2022; Liu et al., 2025). A randomized double-blind controlled trial found that in COPD patients with moderate depression, adding escitalopram to conventional respiratory treatment resulted in a 6.3-point decrease in HAMD-17 score after 6 weeks, significant improvement in quality of life and exercise endurance, while there was no significant change in lung function. This indicates that respiratory improvement drugs exert mental protection effects indirectly through the “function-emotion-cognition” pathway (He et al., 2016).

While most risk factors show consistent associations with poor mental health, age demonstrates an unexpected yet meaningful protective effect in this study. That is, as people age, the risk of developing mental problems such as depression and anxiety decreases for the elderly. This result seems contrary to common sense at first glance, but it is highly consistent with recent international mainstream research and is called the “paradox of better mental health in old age” The FDA's meta-analysis showed that the suicide rate of antidepressant users aged 65 and above was lower than that of the placebo group, while the risk increased in the 18–24 age group (Grunebaum and Mann, 2007). The SSRI/SNRI cohort study confirmed that the intentional self-harm rate was the lowest in the ≥65 age group, and there was no significant difference among different antidepressant drug categories, suggesting that the elderly have better tolerance to antidepressants and a better treatment benefit-risk ratio (Miller et al., 2014). The protective effect of aging on certain mental problems may be the result of the complex interaction of biological, psychological and social factors. At the neurobiological level, age-related brain function remodeling, such as changes in the connections between the prefrontal cortex and the amygdala, prompts older individuals to be better at using emotional regulation strategies like cognitive reappraisal, showing a preference for positive information (Mather and Carstensen, 2005). At the psychological level, older individuals enhance their psychological resilience through accumulated life experiences and wisdom, and can more effectively cope with adversity (Charles and Luong, 2013). At the sociological level, being relieved from work pressure and family responsibilities, reduced role stress, and obtaining higher life satisfaction through downward social comparison. However, this protective effect is influenced by the “healthy survivor” bias and may present an inverted U-shaped curve, being most obvious in “young elderly” and weakened in “old elderly” due to functional decline (Jorm, 2000). The results of this study are consistent with the large-scale meta-analysis by the FDA, confirming that Age has an independent protective effect on mental health issues, providing a theoretical basis of “low risk, high benefit” for the prevention and treatment strategies of depression in the elderly.

Equally intriguing is the finding that frequent pain, which would conventionally be expected to worsen mental health, exhibited a mild but significant protective effect in our study. This finding contrasts interestingly with the traditional notion that “pain leads to depression”, revealing the complex relationship between pain experience and mental health. EU data shows that among new users of antidepressants aged 65 and above, the proportion of those with “pain diagnosis” is significantly lower than that of younger groups. Those with high self-assessed pain often receive lower initial prescriptions and higher discontinuation rates, reflecting the clinical practice tendency of “non-drug first → drug later”, which instead reduces the risk of drug-related mental side effects (Schetz et al., 2024; Romdhani et al., 2023). From a mechanistic perspective, this protective effect is mainly achieved through three pathways: Firstly, elderly individuals who experience frequent pain often develop more effective coping strategies, such as cognitive reappraisal and emotion regulation (Ghandehari et al., 2020); Secondly, pain in the elderly significantly increases the utilization rates of hospitalization, outpatient care, and traditional medicine, while also promoting increased interaction among families, neighbors, and communities, thereby expanding social networks (Pengpid and Peltzer, 2023; Singh et al., 2024; Ai et al., 2023); Thirdly, moderate pain stimulation can upregulate the endogenous opioid system, generating positive emotions similar to the “Runner's high”, and the mindfulness training often included in pain management can also indirectly improve mood (Burgess et al., 2024).

While this study evaluated 12 machine learning algorithms, the primary objective was not methodological competition, but the identification of a clinically viable tool. This study suggests that we should re-examine the clinical significance of pain: when elderly patients complain of “constant pain”, they may be expressing an adaptive state of “having learned to live with pain” rather than seeking help for pain. This subjective experience has an important difference from the objective intensity of pain, and frequent pain combined with good coping strategies may instead become a breeding ground for psychological resilience.

## Limitation

This study has several limitations. Firstly, the cross-sectional design makes it difficult to establish causal relationships; future longitudinal research designs are needed to track the dynamic changes between variables. Secondly, while we defined polypharmacy based on the count of medication categories, we were unable to assess the “appropriateness” of each prescription due to the lack of detailed clinical context in the database. Future clinical cohort studies with access to full prescribing records and indications are needed to differentiate between necessary polypharmacy and potentially inappropriate medications (PIMs). Third, while the study leveraged the ELSA dataset's rigorous design integrating self-reports with nurse verification and standardized coding (BNF), some variables (e.g., medication adherence or symptom recall) still rely on participant reporting, which may introduce recall bias or social desirability bias. Future studies could explore the integration of objective measures (e.g., electronic health records, medication dispensing data, or biological markers) to triangulate self-reported information and further enhance measurement validity. Additionally, the cross-sectional nature of the analysis limits causal inferences; longitudinal designs or nested experimental interventions would help disentangle temporal relationships and mechanisms. In the future, randomized controlled trials based on integrated drug management can be carried out to study the effectiveness of optimizing medication regimens in alleviating mental symptoms, as well as to explore the molecular mechanisms by which different drug combinations affect mental health.

## Conclusion

This study was based on the ELSA data and regarded “whether the doctor diagnosed any emotional, neurological or mental problems” (psyche) as the outcome variable. It systematically explored the associations between the use of antidepressants, age, frequency of pain, smoking history and respiratory improvement drugs and mental problems in the elderly. The results showed that the use of antidepressants was the most significant risk factor; age had a significant protective effect on mental problems, that is, as people age, the risk of depression, anxiety and other mental problems actually decreases in the elderly. Current smoking increases the risk, while respiratory improvement drugs show an independent protective effect; frequent pain (subjective reports) shows a slight protective signal, suggesting the potential role of the “pain–coping–social support” mechanism in the elderly population.

## Data Availability

The original contributions presented in the study are included in the article/Supplementary material, further inquiries can be directed to the corresponding author.

## Supplementary material

Supplementary Figure S1 Feature selection workflow: baseline comparisons and univariate logistic regression that yielded the five variables used for modeling (respiratory-improvement medication, antidepressant use, age, pain frequency, smoking history).

Supplementary Figure S2 Directed weighted network of pairwise model comparisons based on cumulative NRI and IDI. **(A–D)** NRI analysis heatmaps and directed weighted network diagrams for the training set **(A, B)** and the validation set **(C, D)**. **(E–H)** IDI analysis heatmaps and directed weighted network diagrams for the training set **(E, F)** and the validation set **(G, H)**.

Supplementary Figure S3 PD-ICE plots for the five features showing marginal effects on predicted probability in the KNN model.
