# Supplementary figures and images for: Identifying factors and predicting mental health issues in polypharmacy elderly using machine learning: a study based on the English longitudinal study of aging
Source: Front Psychol. 2026 May 15;17:1773958. doi: 10.3389/fpsyg.2026.1773958 (PMC13219346; doi:10.3389/fpsyg.2026.1773958)

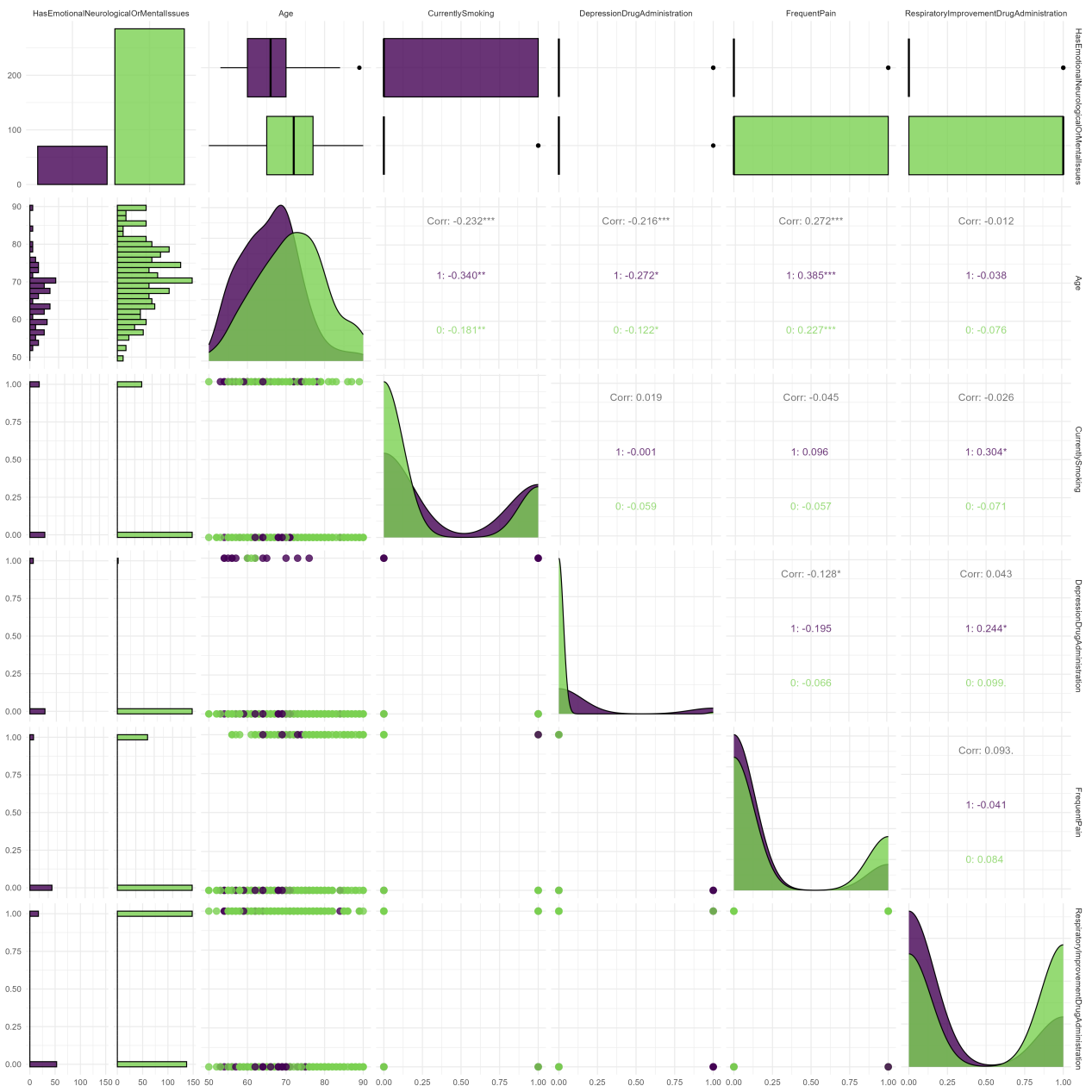

Supplement: Supplementary Figure S1 — Feature selection workflow: baseline comparisons and univariate logistic regression that yielded the five variables used for modeling (respiratory-improvement medication, antidepressant use, age, pain frequency, smoking history). [file Image_1.tiff]

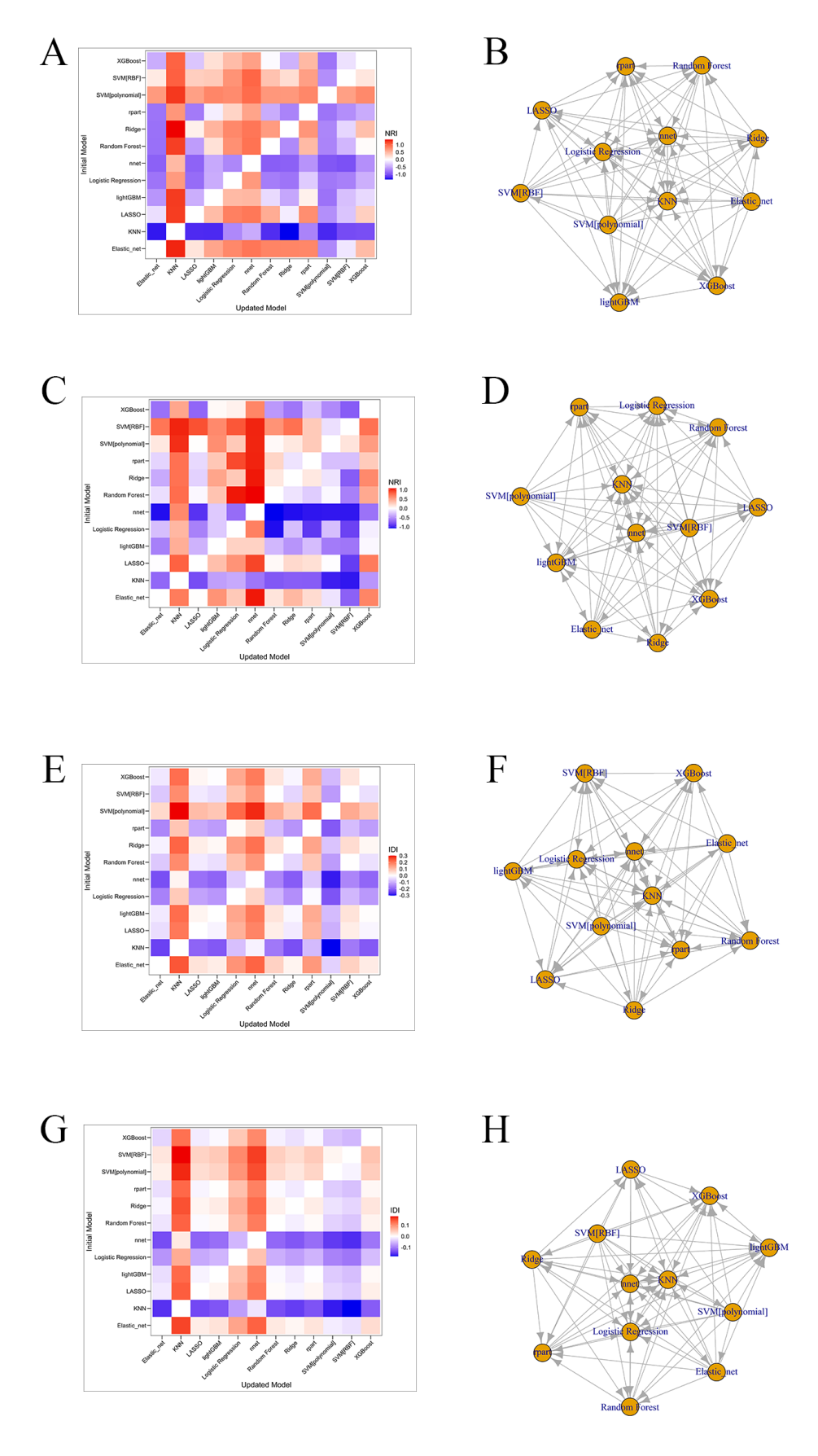

Supplement: Supplementary Figure S2 — Directed weighted network of pairwise model comparisons based on cumulative NRI and IDI. (A–D) NRI analysis heatmaps and directed weighted network diagrams for the training set (A, B) and the validation set (C, D). (E–H) IDI analysis heatmaps and directed weighted network diagrams for the training set (E, F) and the validation set (G, H). [file Image_2.tiff]

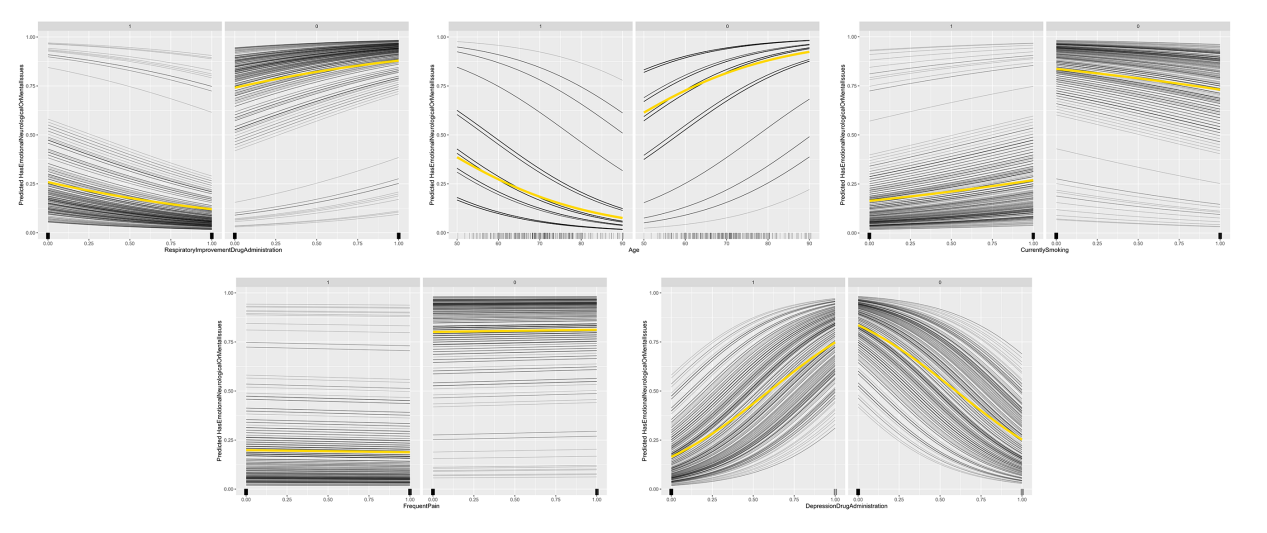

Supplement: Supplementary Figure S3 — PD-ICE plots for the five features showing marginal effects on predicted probability in the KNN model. [file Image_3.tiff]
